# Characterization of Free and Bound Phenolic Acids and Flavonoid Aglycones in *Rosa rugosa* Thunb. Leaves and Achenes Using LC–ESI–MS/MS–MRM Methods

**DOI:** 10.3390/molecules25081804

**Published:** 2020-04-15

**Authors:** Marta Olech, Wioleta Pietrzak, Renata Nowak

**Affiliations:** Chair and Department of Pharmaceutical Botany, Medical University, 1 Chodźki Street, 20-093 Lublin, Poland; wioleta.pietrzak@umlub.pl (W.P.); renata.nowak@umlub.pl (R.N.)

**Keywords:** liquid chromatography mass spectrometry, validation, *Rosaceae*, rose leaves, rose true fruit, flavonoid aglycones, phenolic acids

## Abstract

Fast and reliable determination of polyphenols is a quite common goal during investigation of new plant materials and herbal products, their standardization, quality control, or chemo-taxonomical studies. The aim of this study was to develop and validate methods based on the application of reversed phase liquid chromatography/electrospray ionization triple quadrupole mass spectrometry (LC-ESI-MS/MS) using multiple reaction monitoring (MRM) for comprehensive quantitative and qualitative analysis of phenolic acids and flavonoid aglycones. LC-MS/MS-MRM protocols were applied for the determination of free and bound phenolics in a series of plant samples prepared from leaves and achenes (true fruits) of Japanese rose (*Rosa rugosa* Thunb.). The presence of large amount of phenolic compounds was detected in rose leaves (786.44 µg/g and 14.46 µg/g of phenolic acids and flavonoid aglycones, respectively). Isoferulic acid and five aglycones were revealed for the first time in this plant material. Moreover, 15 phenolic acids and six aglycones were found in the rose achenes, including eight phenolic acids and four aglycones that had not been previously reported in this rose organ. It was shown that leaves and achenes may constitute an industrially relevant source of phenolic compounds for potential commercial use in pharmaceutical, food, or cosmetic industry.

## 1. Introduction

Polyphenols are one of the most valuable and desired ingredients of plants and plant-based products, since they exhibit a wide range of health-beneficial activities, e.g., anti-inflammatory, antioxidant, antiproliferative, chemopreventive, anti-microbial, and enzyme modulating effects [1,2]. They are often the main ingredients responsible for the biological potential of plant species or nature-derived products. The phenolic composition may be influenced by a number of factors, e.g., collection period and place, storage conditions, processing (time, temperature, pressure, solvents), etc. Therefore, qualitative and/or quantitative determination of polyphenolic compounds in raw plant materials, herbal medicines, food, and cosmetic products is often required. 

The total content of polyphenol groups can be evaluated with the use of simple spectrophotometric assays (e.g., with Folin–Ciocalteu reagent, Arnov reagent, AlCl_3_) [3,4,5]. However, in the case of a rich matrix (like plant extracts), colorimetric measurements may be burdened with considerable errors. They are also often insufficient for more demanding approaches. Hence, more sensitive and precise methods of determination are needed. HPLC with UV detection are more robust analytical techniques. They provide separation of analytes and their detection based on the retention time and absorbance. A significant advantage of HPLC-UV is that it enables detection of all of the most intense polyphenols from the sample. However, it has also has some limitations, e.g., relatively low sensitivity, selectivity, and the necessity of separating all the analytes of interest [6,7,8]. Therefore, LC with mass spectrometry (LC-MS) is often preferred for the analyses of complex samples. Liquid chromatography coupled with the triple quadrupole mass spectrometer is an excellent combination for analysis of specific substances in a complex sample. It is selective and provides low levels of detection and quantification [6,9]. Moreover, the analyses are rapid and suitable for routine testing. The development and validation of the qualitative and quantitative LC-MS/MS method is frequently carried out, especially during plant or fungal chemotaxonomic studies, fingerprint analyses, or comparison of samples from different geographical regions [10,11,12]. There are many LC-MS methods created for the analyses of phenolic compounds. Some of them are focused on a given group or subgroup of metabolites, while others cover a broad range of analytes [6,8]. Phenolic acids and flavonoid aglycones are the most commonly determined phenolic compounds found in aerial and underground plant organs. They can occur in a free form and bound with, e.g., simple and complex sugars or other phenolics [9]. For insightful analysis thereof, comprehensive and validated analytical methods covering a broad range of analytes are often required. 

Our previous studies revealed that rugosa rose (*Rosa rugosa* Thunb.) leaves and true fruits (achenes, also called nuts or seeds) are rich in biologically active phenolics [1,13]. However, these rose parts have been poorly studied so far and no insightful analyses of their phenolic acids and flavonoid aglycones have been performed. For example, there is no information about the content of free and bound forms of the above-mentioned phenolic compounds. Determination of the presence, types of conjugates in plant samples, and their quantification provides an important phytochemical information and indicates the possibilities of efficient use of raw material.

The large amounts of rose leaves and achenes constituting an interesting raw material for potential application are obtained from rose plantations every year. Although these rose organs are still being treated as plantation by-products and often discarded without any utilization, the interest in their potential use for commercial purposes (e.g., for preparation of nutraceuticals, functional foods, or pharmaceutical products) has continued to grow in recent years [14,15,16,17,18,19,20,21]. Moreover, rose leaves have been used in folk medicine to cure bronchitis and were found to exhibit anti-angiogenic, anti-inflammatory, and anti-nociceptive activities [22,23]. Recognition of the content of their beneficial metabolites should contribute to the use of these valuable rose materials. Meanwhile, fast and reliable LC-MS protocols have to be applied in the development of herbal products, their standardization, and quality control. Therefore, we aimed at the development and validation of new fast liquid chromatography/electrospray ionization triple quadrupole mass spectrometry (LC-ESI-MS/MS) methods suitable for the determination of phenolic acids and flavonoid aglycones in a series of different samples from the studied plant material. The methods were designed to cover all the most common analytes belonging to the benzoic and cinnamic acid derivatives and few flavonoid aglycone classes. However, we were also interested to include some less common but biologically active compounds (e.g., resorcylic, methoxycinnamic, methoxybenzoic acids, and sakuranetin). To the best of our knowledge, validated LC-MS methods covering a similar set of analytes have not been reported to date. Moreover, qualitative and quantitative contents of free and bound phenolic acids and flavonoid aglycones in rose leaves and achenes have been determined for the first time.

## 2. Results and Discussion

### 2.1. Conditions of the Methods

New LC-MS/MS methods were developed and validated using mixtures of standard compounds. We have previously conducted some LC-DAD analyses of extracts from *R. rugosa* aerial parts. They did not reveal any new phenolic compounds, therefore we decided to develop more sensitive LC-MS methods. The initial LC-DAD work was useful in determining the most abundant species and provided the foundation for the targeted LC-MRM method developed herein. It was shown that 28 phenolic acids or 17 flavonoid aglycones were successfully quantified during a single run time with the use of our optimized LC-MS/MS methods. After obtaining satisfactory results of the validation process, the methods were used for analyses of rose samples. Plant extracts were prepared according to previously optimized and recommended procedures [24]. The newly designed methods were found to be fast, efficient, and solvent-saving approaches.

The method for determination of phenolic acids was developed de novo for 25 compounds (for benzoic and cinnamic acid derivatives). It was designed to comprise all most common and active compounds, often measured in plant or food samples, like gallic, gentisic, caffeic, ferulic, protocatechuic, *p*-coumaric, sinapic, or salicylic acids. However, it also comprised more unique phenolic acids with biological potential (e.g., 3,4-dimethoxycinnamic, veratric acids; Table 1 and Table S1). All compounds of interest are eluted within 15 min of analysis. Analytes with the same optimized multiple reaction monitoring (MRM) transitions can be satisfactorily separated and quantified (e.g., resorcylic acids, ferulic and isoferulic, and coumaric acids; Figure S1). Moreover, the method facilitates separation of *cis*- and *trans*- isomers of caffeic and sinapic acids. LOD (limit of detection) values vary for different phenolic acids from 1.7 ng/mL to 1570 ng/mL for 5-*O*-caffeoylqunic acid and veratric acid, respectively. Similar differences are also observed for the limit of quantification (LOQ) values ranging from 3.5 to 3140 ng/mL. The defined linearity ranges for calibration curves are sufficiently wide for each analyte.

The method for determination of flavonoid aglycones was inspired by an approach previously proposed by Pietrzak et al. [25]; however, the column type, gradient, and method range were modified. The method runtime was shortened, while the identification expanded to include an additional aglycone (Table 2 and Table S2). Moreover, the full validation protocol was accomplished showing that the method meets all requirements for every analyte.

It was shown that 17 flavonoid aglycones can be separated, identified, and quantified using our procedure. Separation of aglycones with the same pseudomolecular ions [M – H]^−^, e.g., luteolin, kaempferol, and sakuranetin with 284.7 *m*/*z* or 3-*O*-methylquercetin, isorhamnetin, and rhamnetin with 314.7 *m*/*z*, is effective using the optimized chromatographic conditions in less than 11 minutes. However, working in the MRM mode (in contrast to the single ion monitoring (SIM) mode) allows distinguishing compounds with the same parent ions but having different fragment ions. In addition, the MS detector and MRM mode ensure high sensitivity. The LOD and LOQ values for aglycones were differentiated, ranging from 1 to 50 ng/mL and from 2 to 70 ng/mL in the case of LOD and LOQ, respectively. The optimized working linearity ranges are quite broad and facilitate convenient work with samples of different concentrations. 

### 2.2. Validation of the Methods

The intra- and inter-day precisions for the standards of phenolic acids and flavonoid aglycones are summarized in Table 1 and Table 2. The percentage CV of intra- and inter-day precision was 0%–5.6% for phenolic acids and was 0.2%–5% for flavonoid aglycones. The coefficient of variation (CV) for instrumental precision was 0.3%–5%. The acceptance criteria are not more than 15% deviation from the nominal value for precision and accuracy. It is desirable that these tolerances be provided for both intraday and inter-day experiments [26]. The validation results indicated that the methods exhibited very good linearity and precision. Endogenous sample components that may interfere with the ionization of our analytes have not been observed.

### 2.3. Identification and Quantification of Polyphenols in *R. rugosa* Leaves and Achenes

The important trend in plant science is to show opportunities and ways to use natural resources more efficiently. That is why different plant by-products are being studied for potential application for food, cosmetic, or pharmaceutical purposes.

The LC-MS methods were used for analysis of phenolic acids (PA) and flavonoid aglycones (FlA) in samples from rugosa rose leaves and achenes. The chemical composition of these rose organs has been poorly studied so far. They are treated mainly as unused plant material obtained in large quantities during cultivation of rose hips and/or petals. In light of some previous studies on the composition and biological activity of rose leaves and hips, it seemed reasonable to know if these rose organs are a valuable source of polyphenols for potential use [15,17,18,19,20]. The analyses were conducted for hydrolyzed and non-hydrolyzed samples to compare qualitatively and quantitatively free and conjugated phenolic compounds in *R. rugosa* leaves (LF) and achenes (true fruits, FRU). Acid and alkaline hydrolyses were performed to release glycoside-linked or ester-linked phenolics, respectively [27].

#### 2.3.1. Phenolic Acids

Leaves of several rose species were previously reported to be a rich source of different classes of polyphenols e.g., flavonoid glycosides, flavonoid aglycones, phenolic acids, and tannins [1,14,28]. Our analysis confirmed the high concentration of PA in this plant material (in total, 786.44 µg/g of dry weight; Table 3), revealing that most of them are glycosidically linked (553.45 µg/g d.w.) or esterified (187.23 µg/g d.w.). The sum of identified free phenolic acids constitutes only 45.76 µg/g of dry plant material. 

Among free PA, *p*-coumaric was found to be the prevailing compound (21.38 µg/g). Several times lower amounts of caffeic, salicylic, protocatechuic, ferulic, gallic, isoferulic, and 4-hydroxy-benzoic were determined (1.40–7.09 µg/g d.w.). Syringic and gentisic acid constituted a small fraction of free PA of the *R. rugosa* leaves (in total 0.55 µg/g d.w.). The presence of vanillic acid in the leaves was confirmed; however, its concentration in the samples was below the quantification limit. 

The acid or alkaline treatment did not reveal any additional PA. On the contrary, syringic, vanillic, and isoferulic acids were not found or were below LOQ in the hydrolyzed samples. The quantitative proportions of glycosidically linked and esterified PA were different than those found in LF-Free. In the LF-AcH sample (containing compounds released from glycosidic linkages), gallic acid was determined in the largest quantities (151.31 µg/g d.w.), followed by a lower amount of *p*-coumaric and protocatechuic acids (118.50 and 91.97 µg/g, respectively). Gentisic acid was one of the dominant glycosidically linked PA (65.77 µg/g), but was less abundant in LF-Free (0.40 µg/g).

In the case of PA detected after alkaline hydrolysis, gallic acid was the dominant compound as well (115.65 µg/g d.w.). *p*-Coumaric and caffeic acids were present in approximately three and six times smaller quantities, respectively. The other PAs (i.e., protocatechuic, gentisic, 4-OH-benzoic, ferulic, isoferulic, and salicylic acids) accounted for 15.84 µg/g of dry leaves. The large amounts of gallic acid observed in the hydrolyzed samples (LF-AcH and LF-AlkH) were probably released from both low and high molecular conjugates and complexes, which are commonly present in roses [29]. 

Gallic, protocatechuic, caffeic, syringic, 4-hydroxy-benzoic, vanillic, gentisic, *p*-coumaric, ferulic, and salicylic acids were previously detected in rugosa leaves [13,30]. However, the content of their free and bound forms have not been reported to date. Moreover, our study revealed the presence of isoferulic acid in rugosa rose leaves and achenes. To date, a small amount of this acid has only been found in *Rosa gizellae* Borb. and *Rosa rubiginosa* L. var comosa leaves [30]. To the best of our knowledge, there is no information about its presence in other roses or rose organs. 

The rose achenes (FRU) were found to be a relatively rich source of phenolics representing different phenolic classes [1]. Karczmarz et al. [21] showed the presence of free and bound PA in *R. canina*, *R. moyesii*, and *R. pendulina* achenes. Our previous study demonstrated the content of gallic, protocatechuic, caffeic, sinapic, *p*-coumaric, and ferulic acids in teas and tinctures from rugosa rose true fruits [1]. However, the presence of phenolic acids in the free or bound forms was not studied. With the use of the optimized LC-MS method, we were now able to analyze a wider range of phenolic acids. The current study has confirmed the presence of all PA that were previously found in *R. rugosa* achenes (Table 3; Figure S2). Additionally, eight phenolic acids (i.e., syringic, p-hydroxy-benzoic, vanillic, gentisic, rosmarinic, isoferulic, salicylic, and 3,4-dimethoxycinnamic) were analyzed qualitatively and quantified for rugosa true fruits for the first time. An example of a chromatogram of bound PA found in the true fruits (achenes) can be found in Supplementary Material (Figure S2).

It was shown that the achenes contained 11.58 µg/g (of dry weight; d.w.) of free phenolic acids. However, a majority of PA is glycosidically linked (38.15 µg/g d.w.). A relatively small portion was released from soluble esters (4.69 µg/g). Gallic and protocatechuic acid prevail in their free form, while the other determined PA are present in the plant material mostly as glycosides. 

Interestingly, vanillic acid was found to be the main component of the free PA fraction in the achenes, followed by an approximately three-fold lower amount of *p*-coumaric and isoferulic acids (5.23, 1.77, and 1.36 µg/g d.w., respectively). In our previous studies, gallic acid was the dominant PA detected in rugosa rose organs [31,32,33]. In turn, its concentration in the achenes is relatively low. 

Isoferulic and vanillic acids were predominant glycoside-bound PA (14.86 and 11.04 µg/g d.w., respectively), followed by lower quantities of *p*-coumaric, ferulic, 4-hydroxy-benzoic and syringic acid (5.73 to 1.14 µg/g d.w.). The content of the other PA found in FRU after acid hydrolysis was relatively small. The hydrolyses revealed the presence of a trace amount of rosmarinic acid, which is not typical of rose. QTRAP working in the MRM mode has high sensitivity, and probably its application facilitated detection of this compound.

*p*-Coumaric and isoferulic acids were found to be the main PA liberated from esters after the alkaline treatment. However, as already mentioned, ester-linked PA constitute a small portion of dry achenes. It was observed that FRU contained only trace amounts of ester-linked caffeic and gentisic acids. In contrast, these compounds are one of the most important PA in leaves.

As a result of our research a detailed report presenting the profile of free and bound PA in rugosa rose achenes, and in roses in general was elaborated. It was shown that many biologically highly active metabolites (e.g., ferulic, isoferulic, caffeic, gallic, *p*-coumaric) can be found in these rose organs.

#### 2.3.2. Flavonoid Aglycones

Different *R. rugosa* organs were previously reported to be a good source of flavonoids, e.g., quercetin, catechin, kaempferol, and their derivatives [14,28,31,32]. Here, we wanted to determine the amount of free and conjugated flavonoid aglycones. 

Our study revealed that a majority of the flavonoid aglycones (FlA) found in the rugosa rose leaves (LF) were in their free form (Table 4). Seven FlA were detected in unhydrolyzed samples from leaves. Quercetin was the dominant compound (6.40 µg/g dry weight), whereas kaempferol and 3-*O*-methylkaempferol were present in over three times lower quantities (2.09 and 1.81 µg/g d.w., respectively). Quercetin and kaempferol were also previously reported as dominant flavonoids in the leaves of *R. canina*, *R. glauca*, *R. moschata*, *R. rubiginosa*, *R. rugosa*, and *R. sempervirens* [13,16,34]. The LC-MS analysis also showed a reasonable amount of free apigenin in the rose leaves. Apigenin and small quantities of its 7-*O*-glucoside were previously detected in rugosa rose leaves [13,35]. Here, we did not confirm the presence of apigenin in the glycosidic form. This is probably related to the aforementioned low concentration of glycoside and its hydrolysis behavior [36]. Free naringenin, eriodictyol, and 3-*O*-methylquercetin were also found in *R. rugosa* leaves. Naringenin and quercetin 3-*O*-methyl ether have not been previously reported in any rose extract. Additionally, eriodictyol was not reported in the rose leaves. Its derivative eriodictyol hexoside has been found only in rose hip [37]. 

Interestingly, the amount of bound FlA in the rose leaves was found to be lower, suggesting that flavonoid glycosides constitute a relatively small proportion of flavonoids in this plant material or are not hydrolyzed in the conditions used. The quantities of bound quercetin and kaempferol were found to be three and four times lower, respectively. On the other hand, isorhamnetin was found only in the acid-hydrolyzed fraction, which may indicate that this compound occurs only in the glycosidic form in *R. rugosa* leaves. The presence of an O-methyl ether kaempferol derivative in rose leaves was reported by Hashidoko [29]. However, our study revealed the presence of 3-*O*-methylkaempferol in rose leaves. Similarly, isorhamnetin and its glucosides were noted in rugosa rose bee pollen [38] and in the leaves, petals, and fruits of other rose species [16,18,39]. However, our report describes the presence of isorhamnetin in *R. rugosa* leaves. 

**Figure 1:**
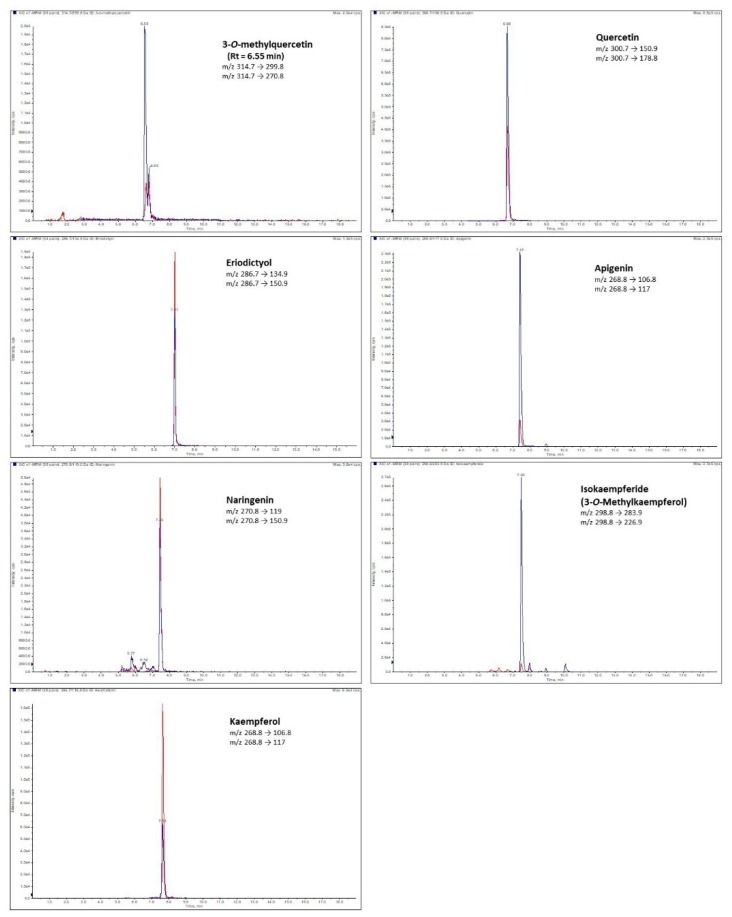
An exemplary LC-ESI-MS/MS chromatograms obtained in MRM mode of free flavonoid aglycones found in *R. rugosa* leaves.

In the case of the rugosa rose achenes (true fruit; FRU), the presence of six flavonoid aglycones in their free forms was revealed (naringenin, kaempferol, quercetin, eriodictyol, isorhamnetin, and taxifolin) (Table 4; Figure 1). Quercetin was the predominant aglycone in FRU, while eriodictyol was the second most abundant (0.13 and 0.03 µg/g d.w., respectively). Taxifolin was found only in the rose achenes, but was not observed either in the free or bound form in the rose leaves. Larger amounts of isorhamnetin and kaempferol were found in the hydrolyzed samples, while the amount of free and bound quercetin was similar in the achenes. It was also observed that eriodictyol occurred in FRU mainly in the free form. The presence of quercetin and kaempferol derivatives was previously noted in rose true fruits (achenes) [13,20]. However, our study is the first one revealing naringenin, eriodictyol, isorhamnetin, and taxifolin in rose achenes. 

## 3. Materials and Methods 

### 3.1. Chemicals

Analytical standards (all with purity ≥ 95%) of caffeic, 5-*O*-caffeoylqunic, gallic, ferulic, isoferulic, homogentisic, protocatechuic, 3-hydroxybenzoic, 4-hydroxybenzoic, rosmarinic, 3,4-dihydroxyphenylacetic, vanillic, syringic, *m*-coumaric, *o*-coumaric, *p*-coumaric, salicylic, 3,4-dimethoxycinnamic, *α*-resorcylic, *β*-resorcylic, *γ*-resorcylic, 3,5-dimethoxybenzoic acid, 3-*O*-methylquercetin, chrysin, isorhamnetin, kaempferol, luteolin, morin, prunetin, rhamnetin, sakuranetin, taxifolin, and LC grade acetonitrile, methanol were purchased from Sigma-Aldrich Fine Chemicals (St. Louis, MO, USA). Gentisic, sinapic, veratric acids, eriodictyol, myricetin and rhamnazin were from ChromaDex (Irvine, USA). Apigenin and naringenin were from Roth (Karlsruhe, Germany) and quercetin was from Fluka (Buchs, Switzerland). LC grade water was prepared using a Millipore Direct-Q3 purification system (Bedford, MA, USA).

### 3.2. Plant Material

Leaves and true fruits (achenes) of *R. rugosa* were collected in Łukówiec (Lublin Voivodeship, Poland) in 2018. The leaves were picked in July. The achenes were collected in August and carefully separated from the red pseudo-fruit (hypanthium). The botanical material was authenticated by Prof. Renata Nowak. A voucher specimen was deposited at the Chair and Department of Pharmaceutical Botany, Medical University of Lublin, Poland (voucher no. R-043/18). The plant material was air-dried at ambient temperature and powdered.

### 3.3. Sample Preparation

At the beginning, the raw materials were pre-treated in a Soxhlet apparatus to remove ballast components. Pulverized achenes were extracted with n-hexane for three days to remove oil. Powdered leaves were extracted with petroleum ether for five days, and then with chloroform for 15 days to remove waxes, chlorophyll, and other ballast substances. After the pre-treatment, the plant residues were dried and divided into 2 g portions, which were extracted three times with 8% (*v*/*v*) methanol at 120 °C using an accelerated solvent extraction system (ASE 150; Dionex Corporation, Sunnyvale, CA, USA). Extracts from the same plant part were combined and the solvent was evaporated in vacuum (40 °C). The residues were eluted with hot water (250 mL per 50 g plant material), cooled, and filtered. Subsequently, fractions of free phenolic acids and aglycones (LF-Free and FRU-Free) were isolated from the water layer by extraction with portions of diethyl ether [24,40]. The ether extracts were concentrated and washed with 5% NaHCO_3_ (*w*/*v*). The alkaline extracts were acidified to pH 2–3 and re-extracted with ether and the final ether layers were dried with anhydrous Na_2_SO_4_.

Bound phenolic acids and aglycones were liberated from the water layers (after removing of free phenolics) using alkaline and/or acid hydrolysis. The acid hydrolysis was performed with concentrated HCl in the following conditions: pH 2–3, 100 °C, 1 h. The alkaline hydrolysis was conducted with NaBH_4_ (0.8 g per 100 mL of extract) and 1% (*w*/*v*) Ba(OH)_2_ at pH 12–13 (100 °C, 15 min.). Both hydrolysates were cooled, filtered, extracted with diethyl ether, and treated in an analogous way as ether fractions for LF-Free and FRU-Free. As a result, fractions AcH and AlkH of phenolic acids released after acid and alkaline hydrolysis were obtained [24,40]. Fraction AcH (after acid hydrolysis) was used to determine the content of bound aglycones. All final ether fractions (Free, AcH, AlkH) were concentrated in a rotary evaporator (at 40 °C) and lyophilized. The dry residues were weighed and stock solutions (50 mg/mL) in methanol were prepared. All extracts and fractions were prepared in triplicate.

The samples were filtered through a hydrophilic PTFE 0.20 μm membrane (Merck, Darmstadt, Germany) syringe filter prior to LC injection.

### 3.4. LC-ESI-MS/MS Analysis of Phenolic Acids and Flavonoid Aglycones

#### 3.4.1. Chromatographic Conditions and Apparatus

Phenolic acids and flavonoid aglycones were determined with the newly developed method using liquid chromatography-electrospray ionization-tandem mass spectrometry (LC-(−)ESI-MS/MS). The methods were developed and optimized for mixtures of standard compounds.

The experiments were carried out using an Agilent 1200 Series HPLC system (Agilent Technologies, Santa Clara, CA, USA) equipped with a binary gradient solvent pump, a degasser, an autosampler, and a column oven coupled with a 3200 QTRAP Mass spectrometer (AB Sciex, Redwood City, CA, USA).

The method for determination of phenolic acids was developed on an Eclipse XDB-C18 column (4.6 × 150 mm, 5-μm particle size; Agilent Technologies, USA). We tested water-methanol and water-acetonitrile mobile phases, a number of column temperatures (20–40 °C), flow rates (200–450 μL/min), and injection volumes (3–10 μL). The best chromatographic separations and peak shapes were obtained using gradient elution with water and acetonitrile (both with 0.1% (*v*/*v*) HCOOH, as solvent A and B, respectively), flow rate 300 μL/min, column temperature maintained at 20 °C, and 3-μL injections. Gradient elution was found to be necessary to achieve satisfactory separation of some analytes with the same MRM pairs (MRM transitions). The best gradient was as follows: 0–2 min 30% (*v*/*v*) B; 4–6 min 40% (*v*/*v*) B; 8–10 min 60% (*v*/*v*) B; 12–16 min 80% (*v*/*v*) B. The total run time was 22 min. 

The method for determination of flavonoid aglycones (FlA) was optimized on a shorter column (Kinetex XB-C18; 2.1 × 50 mm, 1.7-μm particle size; Phenomenex Inc., Torrance, CA, USA) than the one used for PA. As before, we checked the effectiveness of different water-MeOH and water-ACN gradients, column temperatures (20–40 °C), low rates (100–300 μL/min), and injection volumes (2–5 μL). Finally, the chosen parameters were as follows: injection—3 μl, flow rate 200 μL/min, 25 °C, 0.1% (*v*/*v*) HCOOH (solvent A), acetonitrile with 0.1% (*v*/*v*) HCOOH (solvent B), gradient steps: 0–1 min 5% (*v*/*v*) B; 2–5 min 42% (*v*/*v*) B; 6–8 min 55% (*v*/*v*) B; 9–11 min 65% (*v*/*v*) B; 12–13 min 85% (*v*/*v*) B. The total run time was 15 min, which makes the method shorter than that used in our previous studies [25].

#### 3.4.2. Optimization of MS Parameters

The 3200 QTRAP MS/MS system with an ESI source working in the negative mode was used for the mass spectrometric analysis. Nitrogen was used as a nebulizer and collision gas. The optimal mass spectrometer parameters were determined experimentally and were as follow: curtain gas 30 psi, capillary temperature 400 °C, nebulizer gas 60 psi, negative ionization mode source voltage −4500 V for determination of phenolic acids and curtain gas 20 psi, capillary temperature 500 °C, nebulizer gas 30 psi, negative ionization mode source voltage −4500 V for analysis of flavonoid aglycones.

The mass analyzer worked in the multiple reaction monitoring (MRM) scan mode, since it ensures high sensitivity with simultaneous exquisite specificity even when working with complex mixtures. 

The most intense MRM transition was used for quantitative purposes, while two most intense MRM transitions were monitored for the qualitative analysis of each analyte. The compounds were quantified on the basis of peak areas of the most intense MRM transitions and comparison with the results from calibration curves. Standard curves were generated by three repeated injections of known concentrations of standard solutions in the same batch of injections with the biological samples. The Analyst 1.5 software (AB Sciex, Redwood City, CA) was used for data acquisition and analysis. 

Table S1–S2 in the Supplementary Material show a summary of optimized parameters for qualitative analysis of phenolic acids and flavonoid aglycones.

### 3.5. Method Validation

The method was validated for sensitivity, linearity and precision as per the FDA guidelines for the bio-analytical method validation [41].

#### 3.5.1. Linearity

The calibration curves were obtained by analysis of dilutions of standards’ mixtures prepared in 30% (*v*/*v*) solvent B in the case of phenolic acids and 5% (*v*/*v*) B for flavonoid aglycones. Standard solutions at 10 concentration levels were analyzed in triplicate. The calibration curves obtained in the MRM mode were used for analyte quantification. The identified compounds were quantified based on the peak area and comparison with a calibration curve for the corresponding standard. The linearity range for every calibration curve was specified (Table 5 and Table 6).

#### 3.5.2. Limit of Detection (LOD) and Limit of Quantification (LOQ)

The limit of detection (LOD) is defined as the lowest concentration of an analyte that was not necessarily quantifiable but can be reliably detected. The limit of quantification (LOQ) was considered to be the lowest concentration that can be determined with acceptable precision. The LOD and LOQ of the method for analysis of phenolic acids and flavonoid aglycones were determined by measuring the signal-to-noise ratio corresponding to 3 and 10, respectively, injecting a series of diluted solutions of known concentrations of analytes (Table 5 and Table 6).

#### 3.5.3. Repeatability, Intra-Day, and Inter-Day Precision

The precision of the method was validated using a standard solution containing concentrations covering the entire calibration range. The method was validated for repeatability (instrumental precision), as well as intra-day and inter-day precision. 

Repeatability was tested by repeated analysis (n = 10) of a standard solution at three levels (low, medium, and high concentration) on the same day (Table 1 and Table 2).

Precision was determined by calculating the coefficient of variation (CV) of six replicates for three different standard solution concentrations (low, medium, and high) within one sample run (intra-day) and between sample runs (inter-day). The intra-day data reflect the precision of the method in the same conditions within one day and the inter-day precision was verified by repeating the procedure on three different days within one week (Table 1 and Table 2).

#### 3.5.4. Matrix Effect 

The matrix effect was tested according to Bonfiglio et al. [42] using a post-column infusion (20 µL/min) of myricetin, ferulic acid and syringic acid solutions (0.2, 1 and 1 µg/mL, respectively). Extracts’ concentration was 5 mg/mL. Results are presented on Figure S3 and S4 in the supplementary material.

### 3.6. Statistical Analysis

Results were expressed as a mean ± standard deviation (SD) of three replications for each extract tested. Moreover, the coefficient of variation (CV) for instrumental, inter-day, and intra-day precision was determined. All calculations were performed in STATISTICA10.0 (StatSoft). The one-way ANOVA test followed by Duncan test was used to statistical analyze the differences among obtained data. Significance was accepted at *p* < 0.05.

## 4. Conclusions

One of the current trends in plant science is development of effective methods for analysis of complex natural samples. LC-MS/MS working in the MRM mode has some undeniable advantages that make it an excellent tool for analysis of matrix-rich samples, e.g., plant extracts. LC-MS/MS-MRM methods are characterized mainly by high selectivity, high sensitivity, and the possibility of conducting quantitative and qualitative determinations. Hence, this technique is often used in research on plant material, especially requiring low LOD/LOQ levels and high repeatability. 

The newly developed and validated LC-MS/MS-MRM methods facilitate fast simultaneous quantitative and qualitative determination of a broad range of phenolic compounds. Both methods were successfully applied for analyses of rose samples. We believe that they can be also applied for authentication, quality control, or other analyses of raw material and plant-based products. 

Our analyses of the rose organs resulted in detection of sixteen phenolic acids and nine flavonoid aglycones present in the free and bound forms (glycosidically-linked and esterified) in the plant material. Naringenin, eriodictyol, isorhamnetin, 3-*O*-methylquercetin, 3-*O*-methylkaempferol, and isoferulic acid were identified in the *R. rugosa* leaves. Moreover, the presence of naringenin, eriodictyol, isorhamnetin, taxifolin, and a few phenolic acids were shown in the rugosa rose achenes (true fruits). As far as we know, this is the broadest report describing the phenolic composition of the aforementioned rose parts. The rose organs were found to be an interesting source of phytochemicals for potential use in pharmaceutical, food, or cosmetic industry. We hope that the results of our study will contribute to a wider and efficient use of these agricultural wastes. Further studies regarding the biological activity of rose leaves are currently underway in our lab.

## Figures and Tables

**Table 1:** Repeatability (instrumental precision) and intra-day and inter-day precision data of the LC-ESI-MS/MS method for determination of phenolic acids.

Analyte	Nominal Concentration (ng/mL)	Repeatability (% CV^a^) (n = 10)	Precision			
			Inter-day		Intra-day	
			Measured Concentration (ng/mL)	CV^a^ (%)	Measured Concentration (ng/mL)	CV^a^ (%)
Gallic acid	167	4.7	152.5	4.9	156.6	2.1
	666	2.9	651.2	2.0	645	1.6
	3300	1.5	2899	0.9	2912	0.5
5-*O*-caffeoylqunic acid	17.65	2.2	15.0	2.4	14.9	3.3
	176.5	4.0	151.0	3.8	151.	5.3
	3530	1.2	3052	2.1	3077	2.1
Homogentisic acid	167	2.2	156.4	2.2	158.4	0.1
	1665	1.1	1477	0.5	1481	0.1
	11,100	2.4	10475	0.0	10,475	0.0
*α*-Resorcylic acid	347	2.9	337.8	4.7	339.1	6.5
	1735	5.0	1669	1.5	1663.7	2.0
	3470	4.2	3462	1.1	3443.3	0.8
Protocatechuic acid	347	3.6	299	2.5	301.3	3.3
	1700	2.1	1723	1.8	1716	2.4
	17,200	2.5	14,820	1.2	14,850	1.7
*trans*-Caffeic acid	350	2.7	314.7	4.7	322.9	1.8
	700	1.7	597.4	0.4	597.6	0.5
	3500	1.6	3015	2.8	3017	4.0
Syringic acid	666	4.9	589.6	0.8	592	0.7
	3330	2.8	2852	1.8	2855	2.5
	11,100	1.7	9510	1.3	9546	1.6
4-Hydroxybenzoic acid	347	3.5	283.0	4.7	275.8	2.6
	694	4.2	618.7	0.3	619.65	0.3
	3470	2.6	2975	0.5	2983	0.3
Vanillic acid	333	4.7	293.2	4.2	295.15	5.7
	1650	4.8	1439	1.3	1450	0.3
	33,000	2.0	30694	1.7	30,400	0.3
Gentisic acid	333	5.0	306.5	0.9	307.9	0.6
	1670	2.5	1914	2.5	1941	0.6
	3300	2.6	3653	1.9	3692	0.4
*γ*-Resorcylic acid	194	3.6	186.7	1.0	185.65	0.5
	1935	3.4	1873	1.3	1881	1.6
	7740	2.0	6648	1.3	6679	1.4
3-Hydroxybenzoic acid	667	2.3	590	1.6	593.4	1.9
	1334	2.6	1289	1.3	1298	0.1
	6670	1.6	6462	1.4	6487	1.7
*β*-Resorcylic acid	1970	3.4	1760	2.1	1781	0.7
	7860	0.5	6871	0.4	6885	0.1
	19,700	2.1	18180	0.4	18135	0.2
*trans*-Sinapic acid	69.4	4.3	78.3	1.0	78.8	0.0
	694	3.1	669	3.1	681.6	0.1
	3470	0.8	3026	2.7	3060	2.5
4-Hydroxycinnamic acid (*p*-coumaric)	373	3.8	322	2.8	319.1	2.9
	746	3.3	638	0.8	635.1	0.3
	3730	1.9	3476	2.3	3523	0.2
Ferulic acid	347	2.9	319.2	4.9	324.5	5.6
	1735	3.0	1663	1.0	1660	1.3
	11,600	2.1	10,508	1.2	10437	0.5
Rosmarinic acid	357	2.2	377.9	3.4	371.2	2.2
	1790	1.4	1685	1.3	1677	1.5
	7140	1.3	6982	0.8	7005	0.9
Isoferulic acid	343	4.9	299.8	4.8	299	6.8
	3430	1.8	3749	1.0	3738	1.3
	11,400	0.9	11627	0.2	11,625	0.3
3-Hydroxycinnamic acid (*m*-coumaric)	1740	2.0	1613	0.5	1617	0.4
	6940	3.2	6406	0.5	6387	0.1
	17,000	0.9	16,233	0.9	16,150	0.4
Veratric acid	3140	3.0	2931	2.4	2961	2.2
	15,700	1.5	15,403	0.5	15,375	0.5
	31,400	1.0	30547	0.3	30,487	0.1
3.4.5-Trimethoxyphenyl acetic acid	33.3	3.5	29.6	3.1	30	2.4
	333	1.9	362.5	4.0	361.9	5.6
	3330	0.6	3304	2.1	3341	1.3
2-Hydroxybenzoic acid (*o*-coumaric)	182	4.7	178.2	4.4	182.5	1.9
	1820	1.9	1859	3.7	1898	0.3
	3630	2.5	3728	0.5	3740	0.0
3.4-Dimethoxycinnamic acid	167	2.8	158.7	0.5	159	0.4
	1665	1.8	1444	1.7	1450	2.2
	11,100	0.7	10,738	0.8	10,687	0.2
Salicylic acid	330	4.6	362.2	4.4	370.5	2.5
	1650	0.5	1893	2.2	1914	1.5
	3300	2.6	3730	2.2	3773	1.3
3.5-Dimethoxybenzoic acid	1770	3.2	1693	1.3	1700	1.7
	7060	1.9	6416	0.4	6403	0.3
	35,300	3.9	30,072	2.2	30,075	3.2

CV^a^ —coefficient of variation.

**Table 2:** Repeatability (instrumental precision) and intra-day and inter-day precision data of the LC-ESI-MS/MS method for determination of flavonoid aglycones.

Analyte	Nominal Concentration (ng/mL)	Repeatability (% CV) (n = 10)	Precision			
			Inter-day		Intra-day	
			Measured Concentration (ng/mL)	CV (%)	Measured Concentration (ng/mL)	CV (%)
Taxifolin	50	2.7	52	4.0	51.5	3.0
	1000	2.0	1003	0.3	997	0.3
	5000	2.3	4870	2.6	4950	1.0
Myricetin	11	2.0	10.59	3.7	10.81	1.7
	1000	1.0	953	4.7	1004	0.4
	3600	3.2	3450	4.2	3488	3.1
Morin	10	2.2	10.5	5.0	10.2	2.0
	1000	4.7	1042	4.2	1022	2.2
	5000	4.0	4862	2.8	4750	5.0
Eriodictyol	15	3.0	14.3	4.7	15.1	0.7
	1000	3.2	974	2.6	982	1.8
	5000	1.8	4766	4.7	5012	0.2
Luteolin	40	2.5	38	5.0	39.2	2.0
	1000	4.8	988	1.2	989	1.1
	4000	4.1	3850	3.8	3886	2.9
Quercetin	30	2.8	29.8	0.7	28.9	3.7
	500	2.7	489	2.2	475	5.0
	3000	2.2	2876	4.1	2995	0.2
3-*O*-Methylquercetin	15	1.9	14.8	1.3	15.2	1.3
	500	1.7	496	0.8	485	3.0
	3700	1.7	3665	0.9	3710	0.3
Apigenin	12	3.9	12	0.0	11.9	0.8
	1000	3.7	954	4.6	996	0.4
	6000	1.2	5800	3.3	5870	2.2
Naringenin	33	0.5	32.2	2.4	33.5	1.5
	500	2.2	489	2.2	515	3.0
	3000	3.5	3100	3.3	3150	5.0
Kaempferol	33	1.9	33.5	1.5	33.6	1.8
	3000	0.1	2850	5.0	3100	3.3
	20,000	2.0	19,950	0.3	19000	5.0
Isorhamnetin	40	1.7	39.2	2.0	40.5	1.3
	3000	4.5	2890	3.7	2900	3.3
	60,000	2.0	57,000	5.0	61,000	1.7
Isokaempferide(3-*O*-Methylkaempferol)	250	4.6	240	4.0	242	3.2
	3000	1.2	3050	1.7	3100	3.3
	10,000	1.0	9800	2.0	9560	4.4
Rhamnetin	5	1.3	4.9	2.0	5.1	2.0
	200	2.1	198	1.0	205	2.5
	625	1.9	620	0.8	630	0.8
Chrysin	42	3.4	41	2.4	44	4.8
	500	2.7	498	0.4	480	4.0
	2500	2.1	2420	3.2	2600	4.0
Sakuranetin	70	4.3	68	2.9	69	1.4
	1000	4.1	1050	5.0	1020	2.0
	7000	0.9	6800	2.9	6700	4.3
Prunetin	200	3.8	195	2.5	205	2.5
	3000	4.1	2900	3.3	3050	1.7
	20,000	4.8	19,000	5.0	20,500	2.5
Rhamnazin	70	0.2	69	1.4	72	2.9
	3000	3.5	2850	5.0	3050	1.7
	7000	0.3	6800	2.9	7100	1.4

**Table 3:** Content (µg/g of dry plant material) of free and bound phenolic acids found in *R. rugosa* leaves (LF) and true fruits (achenes, FRU); mean values of three replications ± SD. Abbreviations: Free—fraction of free phenolic acids (non-hydrolyzed sample); AcH—fraction of glycosidically linked phenolic acids released with acid hydrolysis; AlkH—fraction of bound (esterified) phenolic acids released with alkaline hydrolysis; BQL—compound detected, but its concentration is below the quantification limit.

Phenolic Acid	RT (min)	LF-Free	LF-AcH	LF-AlkH	FRU-Free	FRU-AcH	FRU-AlkH
		Average ± SD	Average ± SD	Average ± SD	Average ± SD	Average ± SD	Average ± SD
Gallic	5.14	2.08 ^a^ ± 0.03	151.31 ^b^ ± 1.10	115.65 ^c^ ± 0.98	0.27 ^d^ ± 0.01	0.07 ^d^ ± 0.00	0.020 ^d^ ± 0.00
Protocatechuic	5.92	4.00 ^a^ ± 0.26	91.97 ^b^ ± 0.74	8.17 ^c^ ± 0.17	0.83 ^d^ ± 0.01	0.45 ^d^ ± 0.01	0.20 ^d^ ± 0.00
*trans*-Caffeic	6.93	7.09 ^a^ ± 0.05	62.97 ^b^ ± 1.74	17.23 ^c^ ± 0.11	0.01 ^d^ ± 0.00	0.30 ^d^ ± 0.01	BQL
*cis*-Caffeic	7.10	0.06 ± 0.00	0	0	0	0	0
Syringic acid	7.20	0.15 ^a^ ± 0.00	BQL	0	0.56 ^a^ ± 0.01	1.14 ^a^ ± 0.01	0.09 ^a^ ± 0.00
4-Hydroxy-benzoic	7.33	1.40 ^a^ ± 0.08	23.6 ^b^ ± 0.26	BQL	0.77 ^a^ ± 0.02	1.37 ^a^ ± 0.01	0.14 ^a^ ± 0.00
Vanillic	7.45	BQL	0	0	5.22 ^a^ ± 0.10	11.03 ^b^ ± 0.19	0.31 ^c^ ± 0.00
Gentisic	7.79	0.40 ^a^ ± 0.00	65.77 ^b^ ± 0.90	3.06 ^c^ ± 0.04	0.01 ^a^ ± 0.00	0.04 ^a^ ± 0.00	BQL
*trans*-Sinapic	9.23	0	0	0	0.05 ^a^ ± 0.00	0.07 ^a^ ± 0.00	0.02 ^a^ ± 0.00
*cis*-Sinapic	9.78	0	0	0	0.03 ^a^ ± 0.00	0.14 ^a^ ± 0.00	0.03 ^a^ ± 0.00
*p*-Coumaric	9.33	21.38 ^a^ ± 0.24	118.50 ^b^ ± 2.12	38.50 ^c^ ± 0.56	1.77 ^d^ ± 0.04	5.73 ^e^ ± 0.26	1.73 ^d^ ± 0.05
Ferulic	9.89	2.20^ac^ ± 0.05	15.23 ^b^ ± 0.25	3.83 ^a^ ± 0.08	0.61 ^c^ ± 0.01	2.50^ad^ ± 0.01	0.40 ^c^ ± 0.01
Rosmarinic	10.23	0	0	0	0	0.01 ^a^ ± 0.00	0.01 ^a^ ± 0.00
Isoferulic	10.56	1.83 ^a^ ± 0.04	BQL	BQL	1.36 ^a^ ± 0.03	14.86 ^b^ ± 0.21	1.52 ^a^ ± 0.02
3,4-Dimethoxycinnamic	13.18	0	0	0	0.05 ^a^ ± 0.00	0.39 ^a^ ± 0.00	0.21 ^a^ ± 0.00
Salicylic	14.19	5.17 ^a^ ± 0.18	24.10 ^b^ ± 0.36	0.78 ^c^ ± 0.02	0.02 ^c^ ± 0.00	0.03 ^c^ ± 0.00	0.01 ^c^ ± 0.00
**SUM**		45.76	553.45	187.23	11.58	38.15	4.69

Values are presented in mean ± standard deviation (n = 3) and evaluated by one-way ANOVA test (post test: Duncan test). Different superscript letters (a–e) in the same rows denotes significant differences at *p* < 0.05.

**Table 4:** Content of free and bound flavonoid aglycones in *R. rugosa* leaves (LF) and true fruits (FRU). Mean values of three replicate assays with standard deviation. Abbreviations: Free—fraction of free aglycones (non-hydrolyzed sample); AcH—fraction of bound aglycones (sample after acid hydrolysis); BQL—compound detected, but its concentration is below the quantification limit.

	LF-Free	LF-AcH	FRU-Free	FRU-AcH
	µg/g Dry Plant Material		µg/g Dry Plant Material	
Taxifolin	-	-	<BQL	-
Eriodictyol	0.07 ^a^ ± 0.00	-	0.03 ^a^ ± 0.00	<BQL
Quercetin	6.3 9^a,^* ± 0.15	1.94 ^b,^* ± 0.02	0.13 ^b,^* ± 0.00	0.11 ^b,^* ± 0.01
3-*O*-Methylquercetin	0.04 ± 0.00	-	-	-
Apigenin	1.27 ± 0.01	-	-	-
Naringenin	0.31 ± 0.00	-	< BQL	-
Kaempferol	2.09 ^a^ ± 0.03	0.51 ^a,b^ ± 0.01	< BQL	0.03 ^b^ ± 0.00
Isorhamnetin	-	0.03 ^a^ ± 0	< BQL	0.05 ^a^ ± 0.00
3-*O*-Methylkaempferol	1.81 ± 0.01	-	-	-

Values are presented in mean ± standard deviation (n = 3) and evaluated by one-way ANOVA test (post test: Duncan test). Different superscript letters (a,b) in the same rows denotes significant differences at *p* < 0.05 (“*”—*p* < 0.001).

**Table 5:** Analytical parameters of LC-MS/MS quantitative method for determination of phenolic acids.

Compound	LOD (ng/mL)	LOQ (ng/mL)	R^2^	Linearity Range (ng/mL)
Gallic acid	33.3	95	0.9982	167–3300
5-*O*-caffeoylqunic acid	1.7	3.5	0.9993	3.5–3530
Homogentisic acid	33.3	68	0.9998	68–11100
*α*-Resorcylic acid	174	347	0.9990	347–3470
Protocatechuic acid	68	174	0.9996	174–17200
*trans*-Caffeic acid	60	160	0.9990	175–3500
Syringic acid	167	666	0.9993	666–11100
4-OH-benzoic acid	17.4	34.7	0.9993	69.4–3470
Vanillic acid	100	250	0.9997	330–33000
Gentisic acid	16.7	67	0.9991	67–1670
*γ*- Resorcylic acid	38.7	194	0.9978	194–7740
3-Hydroxybenzoic acid	33.3	334	0.9994	334–6670
*β*- Resorcylic acid	39.3	78.6	0.9992	197–19700
*trans*-Sinapic acid	17.4	69.4	0.9999	69.4–3470
*trans*-*p*-Coumaric acid	18.7	74.6	0.9996	187–3730
*trans*-Ferulic acid	17.4	34.7	0.9994	69.4–11600
Rosmarinic acid	7.1	17.9	0.9994	19.9–7140
*trans*-Isoferulic acid	17.2	686	0.9997	343–11400
*trans*-*m*-Coumaric acid	17	68	0.9993	174–17000
Veratric acid	1570	3140	0.9980	3140–31400
3,4,5-Trimethoxyphenylacetic acid	3.3	9.5	1.0000	16.7–3330
*trans*-*o*-Coumaric acid	7.3	18.1	0.9996	18.1–1820
3,4-Dimethoxycinnamic acid	3.3	33.3	0.9994	66.7–11100
Salicylic acid	3.3	16.5	0.9989	16.5–1650
3,5-Dimethoxybenzoic acid	176.5	470.7	0.9996	706–35300

**Table 6:** Analytical parameters of LC-MS/MS quantitative method for determination of flavonoid aglycones.

Compound	LOD (ng/mL)	LOQ (ng/mL)	R^2^	Linearity Range (ng/mL)
Taxifolin	20	50	0.9986	50–5000
Myricetin	3	5	0.9991	11–3600
Morin	2	4	0.9985	10–5000
Eriodictyol	5	15	0.9980	15–5000
Luteolin	25	40	0.9970	40–4000
Quercetin	2	3	0.9975	30–3000
3-*O*-Methylquercetin	1	2	0.9989	15–3700
Apigenin	3	4	0.9987	12–6000
Naringenin	25	33	0.9990	33–3000
Kaempferol	20	33	0.9986	33–20000
Isorhamnetin	12	24	0.9975	40–60000
Isokaempferide	1	2	0.9993	250–10000
Rhamnetin	2	5	0.9984	5–625
Chrysin	25	40	0.9959	42–2500
Sakuranetin	34	46	0.9990	70–7000
Prunetin	50	75	0.9985	200–20000
Rhamnazin	50	70	0.9991	70–7000

## Supplementary Materials

The following are available online at https://www.mdpi.com/1420-3049/25/8/1804/s1, **Table S1**. The optimized LC-MS parameters for qualitative analysis of phenolic acids; **Table S2**. The optimized LC-MS parameters for qualitative analysis of flavonoid aglycones; **Figure S1**. LC-MS/MS-MRM chromatogram of phenolic acids standards sharing the same MRM transitions; *m*/*z* 166.8–107.9—homogentisic and vanillic acid; *m*/*z* 152.8–109—resorcylic acids; *m*/*z* 136.8–93—hydroxybenzoic acids; *m*/*z* 162.8–119—coumaric acids; *m*/*z* 192.8–133.9—ferulic and isoferulic acid; *m*/*z* 180.7–136.9—veratric and 3,5-dimethoxybenzoic acid. Rt values as given in Table S1; **Figure S2**. An exemplary LC-MS/MS-MRM chromatogram of bound phenolic acids found in *R. rugosa* true fruits (achenes); **Figure S3.** MRM chromatograms showing the effect of endogenous sample components that interfere with the ionization of ferulic acid (blue) and syringic acid (red); **Figure S4.** MRM chromatograms showing the effect of endogenous sample components that interfere with the ionization of myricetin.
