# Supplementary material for: Characterization of Free and Bound Phenolic Acids and Flavonoid Aglycones in Rosa rugosa Thunb. Leaves and Achenes Using LC–ESI–MS/MS–MRM Methods
Source: Molecules. 2020 Apr 15;25(8):1804. doi: 10.3390/molecules25081804 (PMC7221549; doi:10.3390/molecules25081804)

SUPPLEMENTARY MATERIAL

Characterization of Free and Bound Phenolic Acids and Flavonoid Aglycones in *Rosa rugosa* Thunb. Leaves and Achenes Using LC–ESI–MS/MS–MRM Methods

Marta Olech*, Wioleta Pietrzak, Renata Nowak

Chair and Department of Pharmaceutical Botany, Medical University, 1 Chodźki Street, 20-093 Lublin, Poland

* Corresponding author. Tel.: +48 81 448 70 63; fax : +48 81 448 70 60;

E-mail address: marta.olech@umlub.pl

Keywords: liquid chromatography mass spectrometry; validation; *Rosaceae*; rose leaves; rose true fruit; flavonoid aglycones; phenolic acids

Table S1. The optimized LC-MS parameters for qualitative analysis of phenolic acids.

Compound	Retention time [min]	Q1 [m/z]	Q3 [m/z]	DP[a] [V]	EP[b] [V]	CEP[c] [V]	CE[d] [eV]	CXP[e] [V]
Gallic acid	5.14	168.7	78.9	-35	-3	-12	-36	0
			124.9*	-35	-3	-12	-14	0
5-*O*-caffeoylqunic acid	5.19	352.9	190.8*	-35	-4.5	-16	-24	-2
			84.9	-35	-4.5	-16	-60	0
Homogentisic acid	5.27	166.8	123	-25	-5	-12	-12	0
			108*	-25	-5	-12	-36	0
α-resorcylic acid	5.47	152.8	109	-30	-10.5	-10	-12	0
			64.9*	-30	-10.5	-10	-20	0
Protocatechuic acid	5.91	152.9	80.9	-55	-1	-10	-26	0
			107.8*	-55	-1	-10	-38	0
trans-Caffeic acid	6.92	178.7	88.9	-30	-6.5	-12	-46	0
			134.9*	-30	-6.5	-12	-16	0
cis-Caffeic acid	7.10	178.7	88.9	-30	-6.5	-12	-46	0
			134.9	-30	-6.5	-12	-16	0
Syringic acid	7.19	196.9	122.8	-30	-9	-12	-24	0
			181.9*	-30	-9	-12	-12	-2
4-hydroxybenzoic acid	7.32	136.8	92.9*	-30	-7	-10	-18	0
Vanilic acid	7.45	166.8	107.9*	-35	-4	-12	-18	0
			123	-35	-4	-12	-12	0
Gentisic acid	7.78	152.9	80	-70	-4	-16	-110	0
			107.9*	-70	-4	-16	-52	0
γ- resorcylic acid	7.99	152.8	108.9	-35	-3	-10	-14	0
			65*	-35	-3	-10	-30	0
3-hydroxybenzoic acid	8.10	136.9	93*	-35	-4	-16.72	-16	-2
			75	-35	-4	-16.72	-48	0
β- resorcylic acid	8.92	152.8	65*	-35	-2	-10	-24	0
			108.9	-35	-2	-10	-12	0
trans-Sinapic acid	9.23	222.8	121*	-35	-8.5	-10	-36	0
			148.9	-35	-8.5	-10	-20	0
cis-Sinapic acid	9.77	222.8	121	-35	-8.5	-10	-36	0
			148.9	-35	-8.5	-10	-20	0
trans-*p*-Coumaric acid (*trans*-4-hydroxycinnamic)	9.33	162.8	93	-30	-8	-12	-44	0
			119*	-30	-8	-12	-14	0
trans-Ferulic acid	9.88	192.8	133.9*	-25	-11.5	-14	-16	0
			177.9	-25	-11.5	-14	-12	-2
Rosmarinic acid	10.23	358.7	132.6	-50	-5	-26	-44	0
			160.8*	-50	-5	-26	-20	-2
trans-Isoferulic acid	10.55	192.8	133.9*	-30	-9.5	-14	-16	-2
			177.9	-30	-9.5	-14	-12	-2
m-Coumaric acid (*trans*-3-Hydroxycinnamic acid)	10.69	162.8	91	-35	-4.5	-12	-36	0
			119*	-35	-4.5	-12	-14	0
Veratric acid	10.82	180.7	121.9	-35	-6	-14	-18	0
			136.9*	-35	-6	-14	-12	0
3,4,5-trimethoxyphenylacetic acid	10.97	224.9	166.1*	-20	-3.5	-10	-12	-2
			180.8	-20	-3.5	-10	-10	-2
o-Coumaric acid (*trans*-2-Hydroxycinnamic acid)	11.87	162.8	119*	-25	-5	-10	-14	0
			93	-25	-5	-10	-46	0
3,4-dimethoxycinnamic acid	13.18	206.9	103.1	-30	-10	-16	-16	0
			163*	-30	-10	-16	-12	-2
Salicylic acid	14.18	136.9	75	-35	-4	-10	-48	0
			93*	-35	-4	-10	-16	-2

3,5-dimethoxybenzoic acid	14.53	180.8	136.9*	-35	-4	-14	-12	-2
			122	-35	-4	-14	-20	0

[a]DP- Declustering Potential; - [b]EP- Entrance Potential; [c]CEP- Cell Entrance Potential;
[d]CE- Collision Energy; [e]CXP- Collision Cell Exit Potential; *Quantification ion

Table S2. The optimized LC-MS parameters for qualitative analysis of flavonoid aglycones.

Compound	Retention time [min]	Q1 [m/z]	Q3 [m/z]	DP[a] [V]	EP[b] [V]	CEP[c] [V]	CE[d] [eV]	CXP[e] [V]
Taxifolin	5.61	302.7	124.9*	-45	-3.5	-18	-26	0
			284.8	-45	-3.5	-18	-14	-4
Myricetin	5.95	316.7	136.9*	-55	-9	-14	-32	0
			150.9	-55	-9	-14	-26	0
Morin	6.25	300.7	124.9*	-50	-3.5	-20	-24	0
			106.9	-50	-3.5	-20	-30	0
Luteolin	6.54	284.7	132.9*	-75	-9	-18	-38	0
			150.9	-75	-9	-18	-26	0
3-*O*-Methylquercetin	6.55	314.7	299.8*	-55	-9.5	-22	-18	-4
			270.8	-55	-9.5	-22	-26	-4
Quercetin	6.67	300.7	150.9*	-60	-2.5	-12	-26	0
			178.8	-60	-2.5	-12	-20	-2
Eriodictyol	7.01	286.7	134.9*	-45	-6	-12	-32	0
			150.9	-45	-6	-12	-18	-2
Apigenin	7.42	268.8	117*	-70	-9.5	-12	-44	0
			106.8	-70	-9.5	-12	-34	0
Naringenin	7.45	270.8	119*	-50	-11.5	-12	-34	0
			150.9	-50	-11.5	-12	-22	0
Isokaempferide (3-*O*-Methylkaempferol)	7.49	298.8	283.9*	-50	-4.5	-12	-18	-4
			226.9	-50	-4.5	-12	-28	-2
Kaempferol	7.62	284.7	116.8*	-70	-5	-12	-46	0
			93	-70	-5	-12	-52	0
Isorhamnetin	7.7	314.7	299.7	-65	-2.5	-26	-20	-4
			150.9*	-65	-2.5	-26	-30	0
Rhamnetin	8.7	314.7	165*	-60	-5.5	-18	-24	0
			120.9	-60	-5.5	-18	-36	0
Chrysin	9.81	252.8	208.9*	-80	-10	-14	-22	-2
			142.9	-80	-10	-14	-26	0
Sakuranetin	9.9	284.7	118.9*	-60	-5.5	-12	-34	0
			164.8	-60	-5.5	-12	-20	-2
Prunetin	10.05	282.8	267.7	-55	-12	-18	-20	-4
			238.7*	-55	-12	-18	-26	-2
Rhamnazin	10.45	328.7	270.8*	-70	-3	-28	-26	-2
			313.8	-70	-3	-28	-14	-4

[a]DP- Declustering Potential; - [b]EP- Entrance Potential; [c]CEP- Cell Entrance Potential;
[d]CE- Collision Energy; [e]CXP- Collision Cell Exit Potential; *Quantification ion

Figure S1. LC-MS/MS-MRM chromatogram of phenolic acids standards sharing the same MRM transitions; m/z 166.8 → 107.9 - homogentisic and vanilic acid; m/z 152.8 → 109 – resorcylic acids; m/z 136.8 → 93 – hydroxybenzoic acids; m/z 162.8 → 119 – coumaric acids; m/z 192.8 → 133.9 – ferulic and isoferulic acid; m/z 180.7→ 136.9 – veratric and 3,5-dimethoxybenzoic acid. Rt values as given in Table S1.

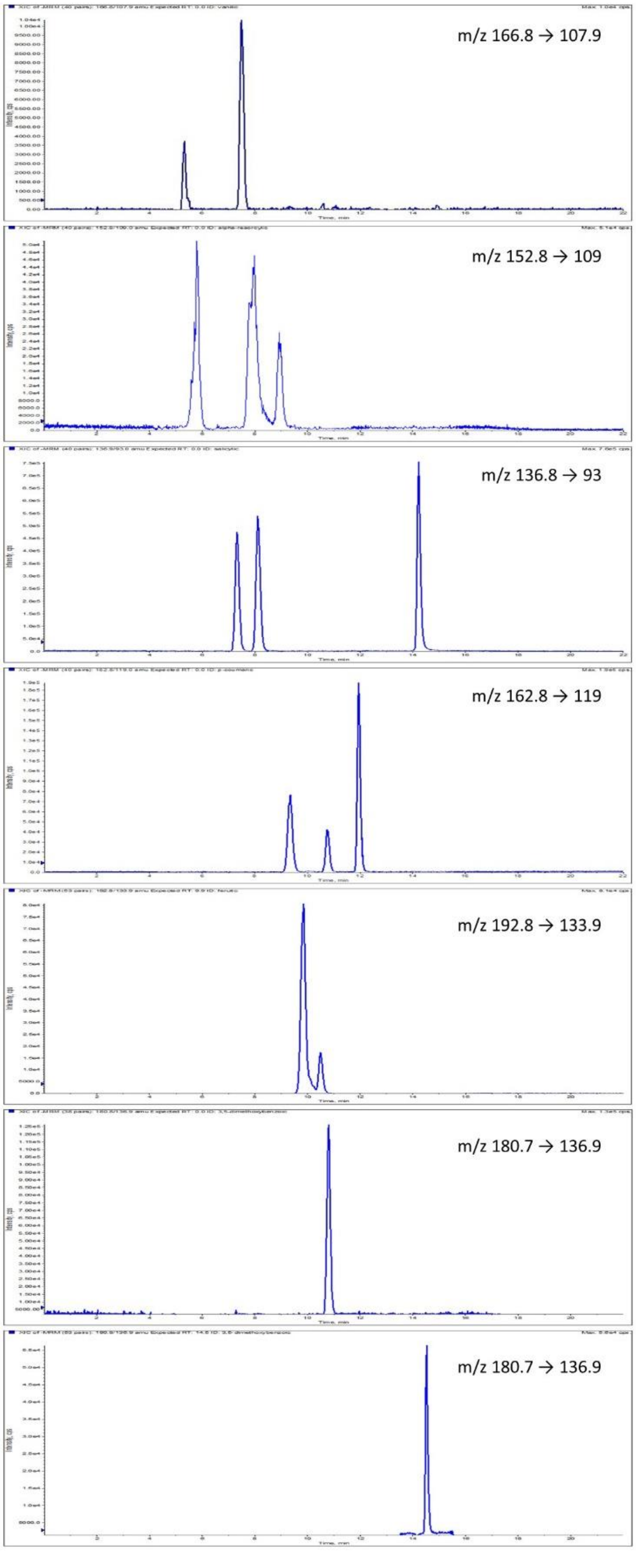

Figure S2. An exemplary LC-MS/MS-MRM chromatogram of bound phenolic acids found in *R. rugosa* true fruits (achenes).

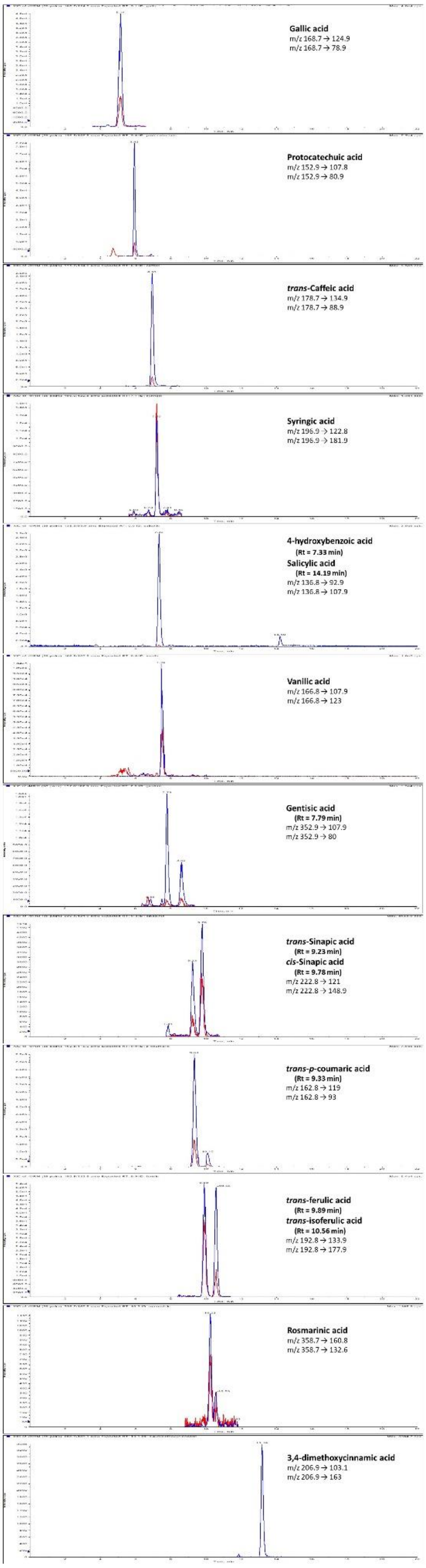

Figure S3. MRM chromatograms showing the effect of endogenous sample components that interfere with the ionization of ferulic acid (blue) and syringic acid (red).

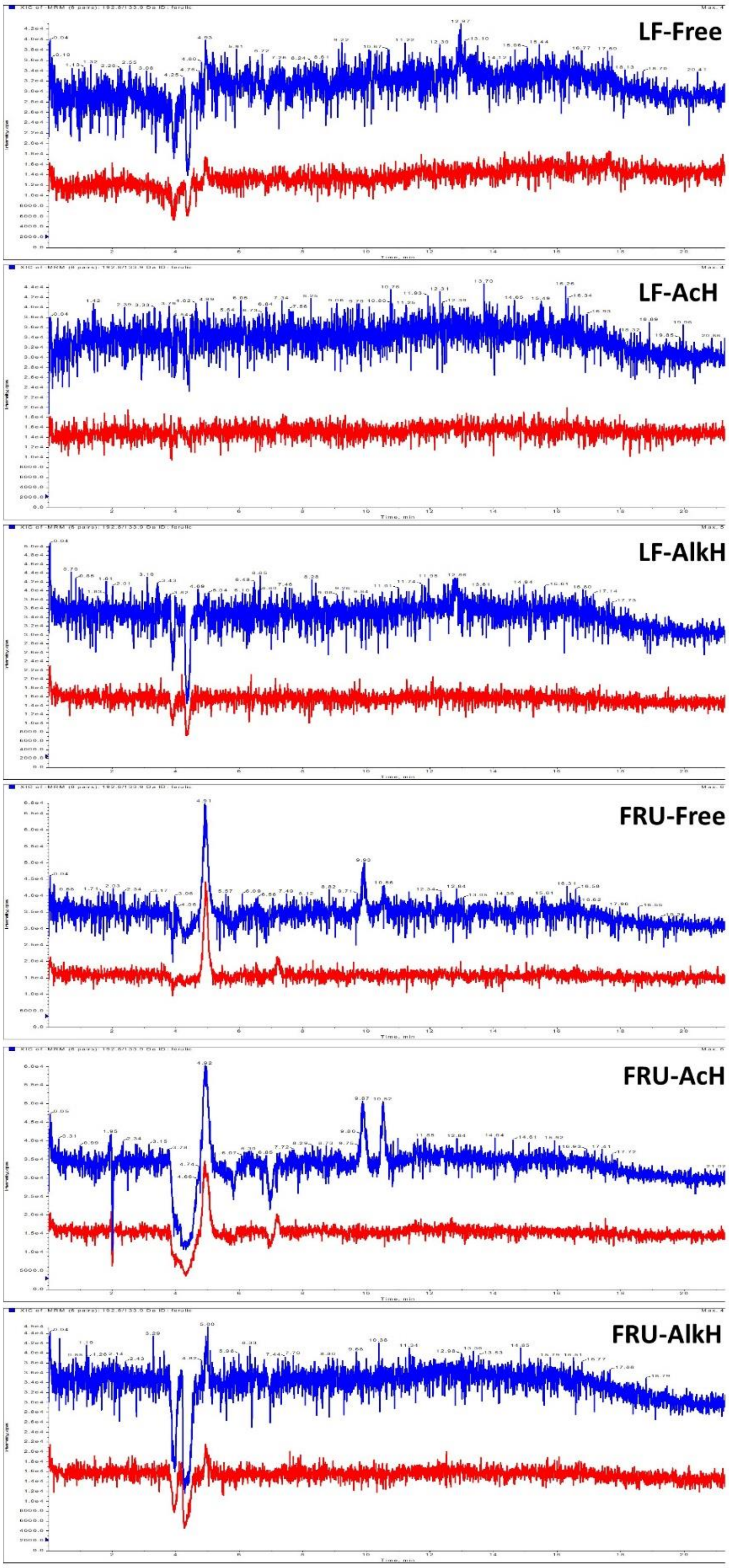

Figure S4. MRM chromatograms showing the effect of endogenous sample components that interfere with the ionization of myricetin.

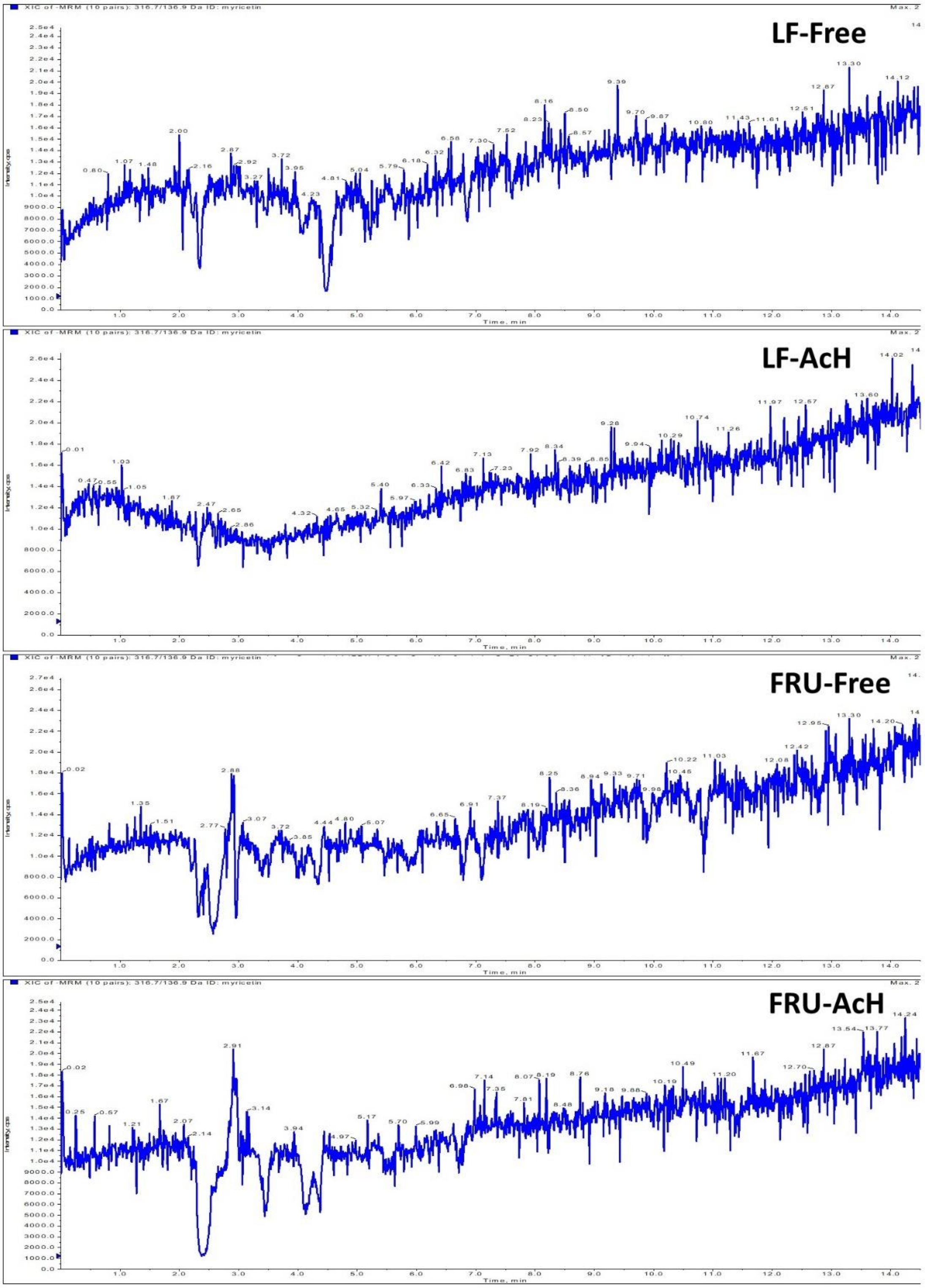

Supplement: Supplementary file 1 [file molecules-25-01804-s001.zip › molecules-765063-supplementary/Supplementary Material Molecules.pdf]
