# Supplementary figures and images for: Characterization of Free and Bound Phenolic Acids and Flavonoid Aglycones in Rosa rugosa Thunb. Leaves and Achenes Using LC–ESI–MS/MS–MRM Methods
Source: Molecules. 2020 Apr 15;25(8):1804. doi: 10.3390/molecules25081804 (PMC7221549; doi:10.3390/molecules25081804)

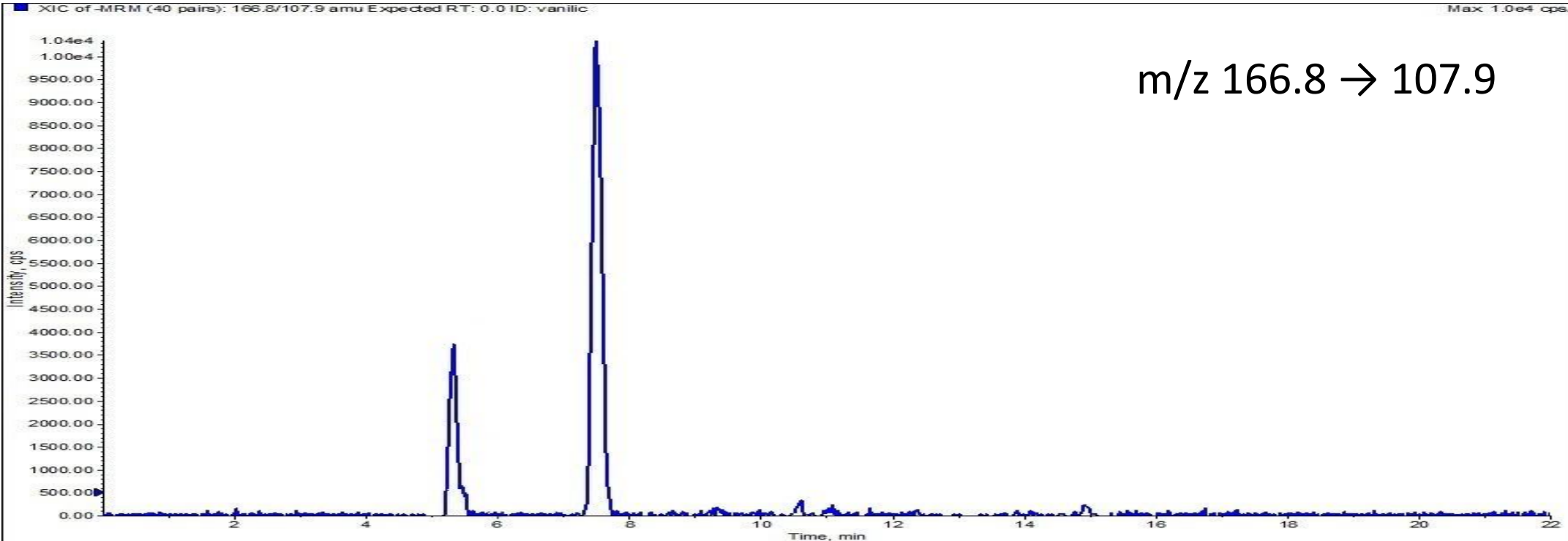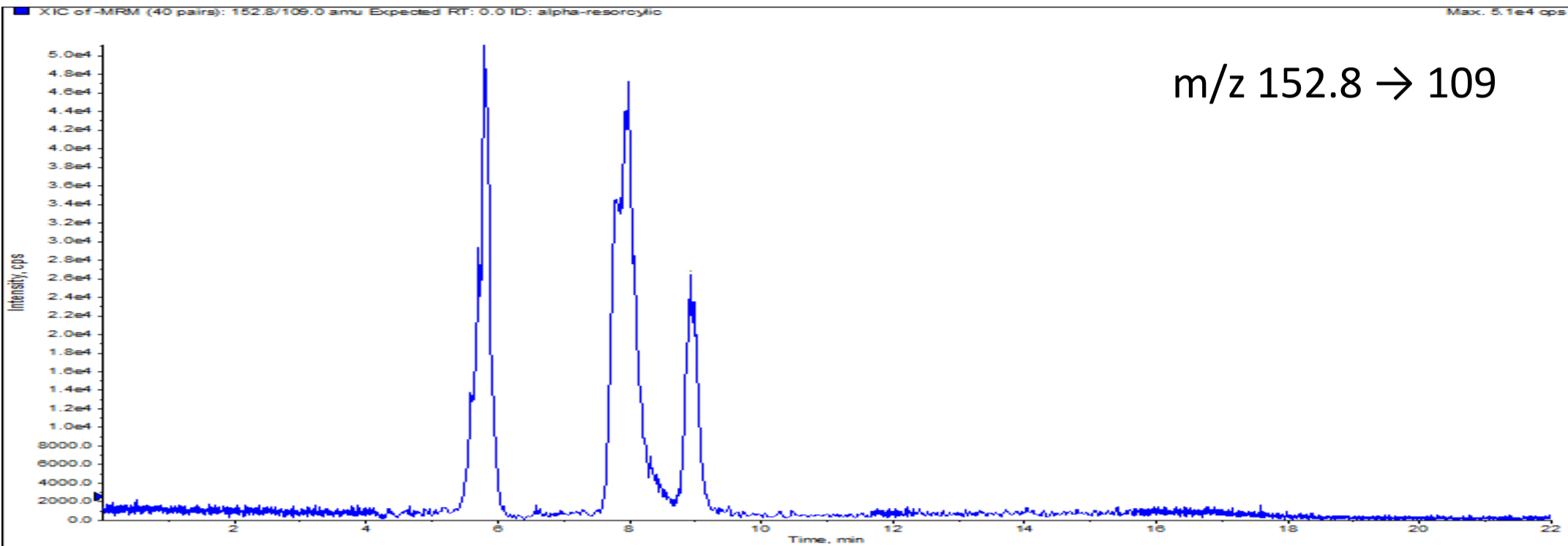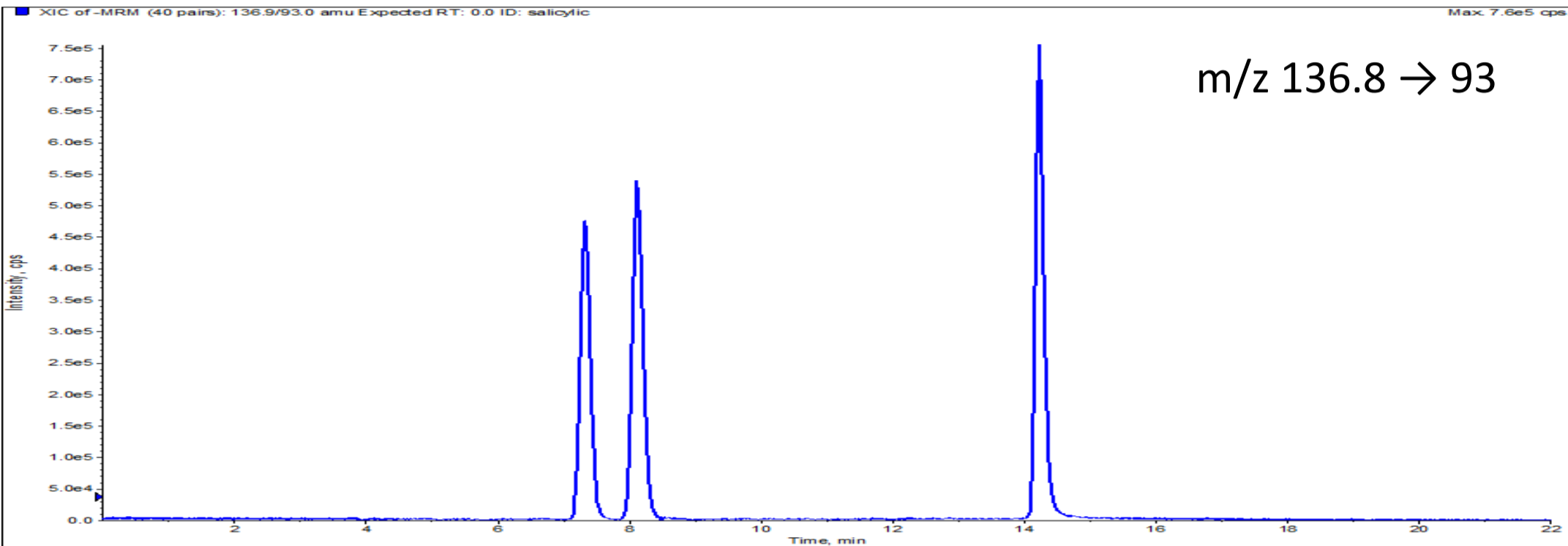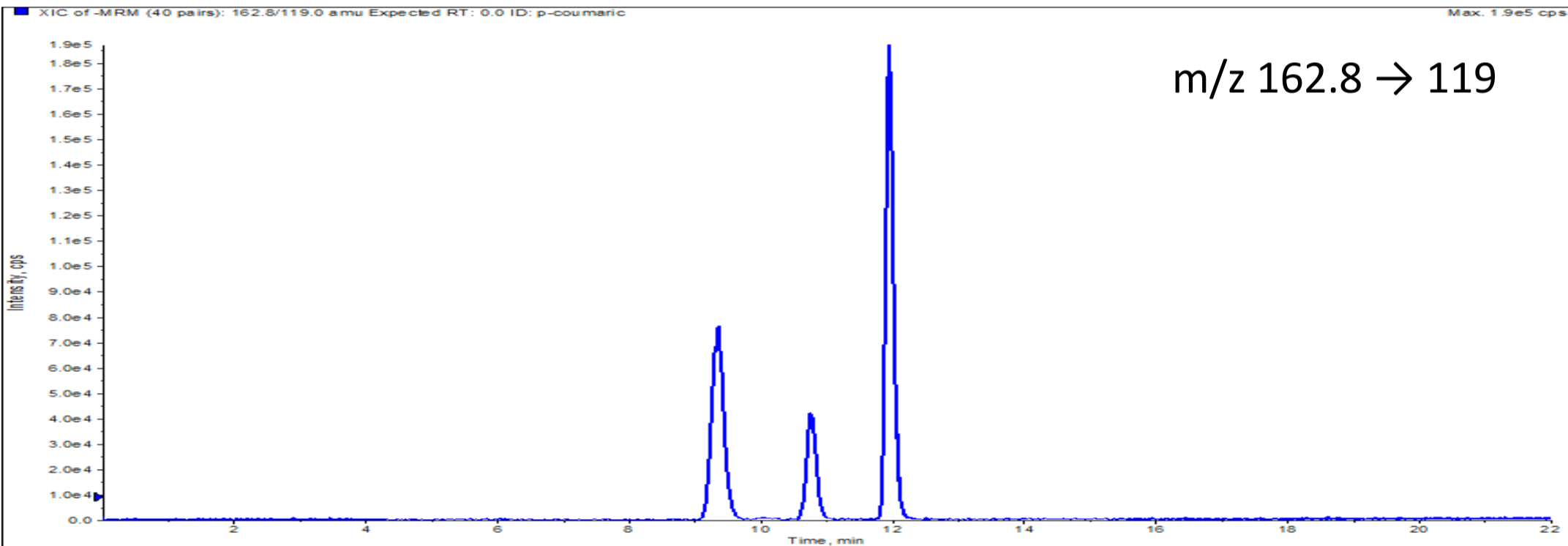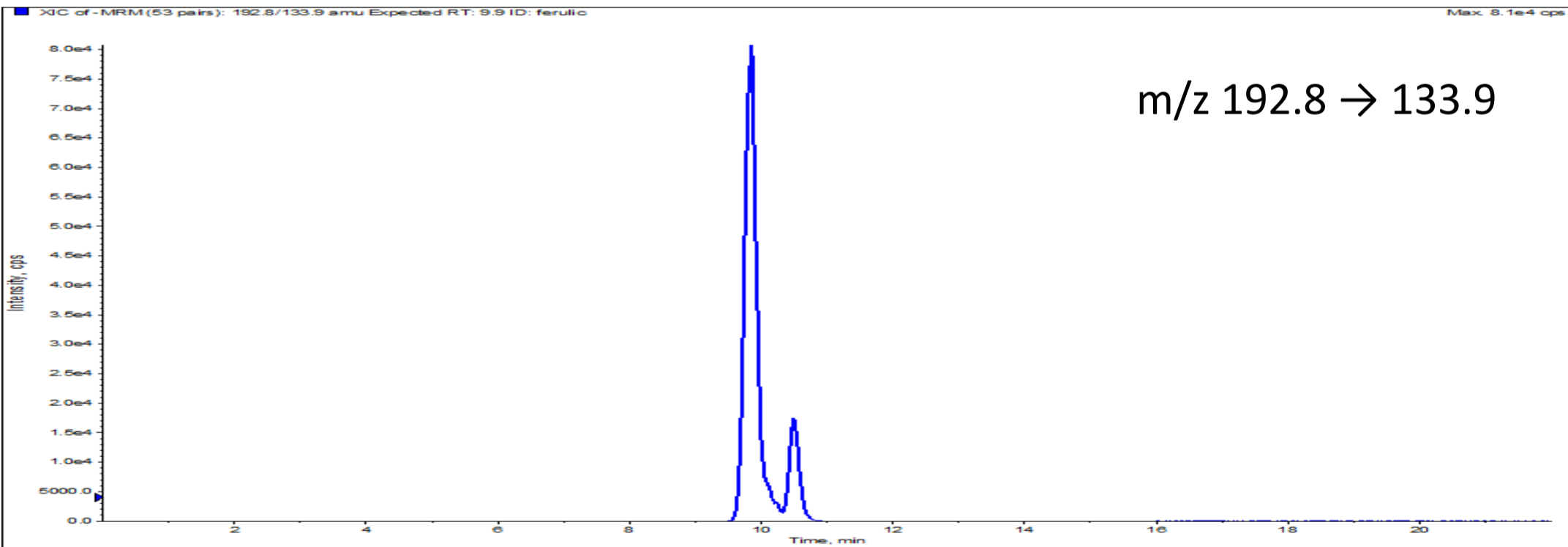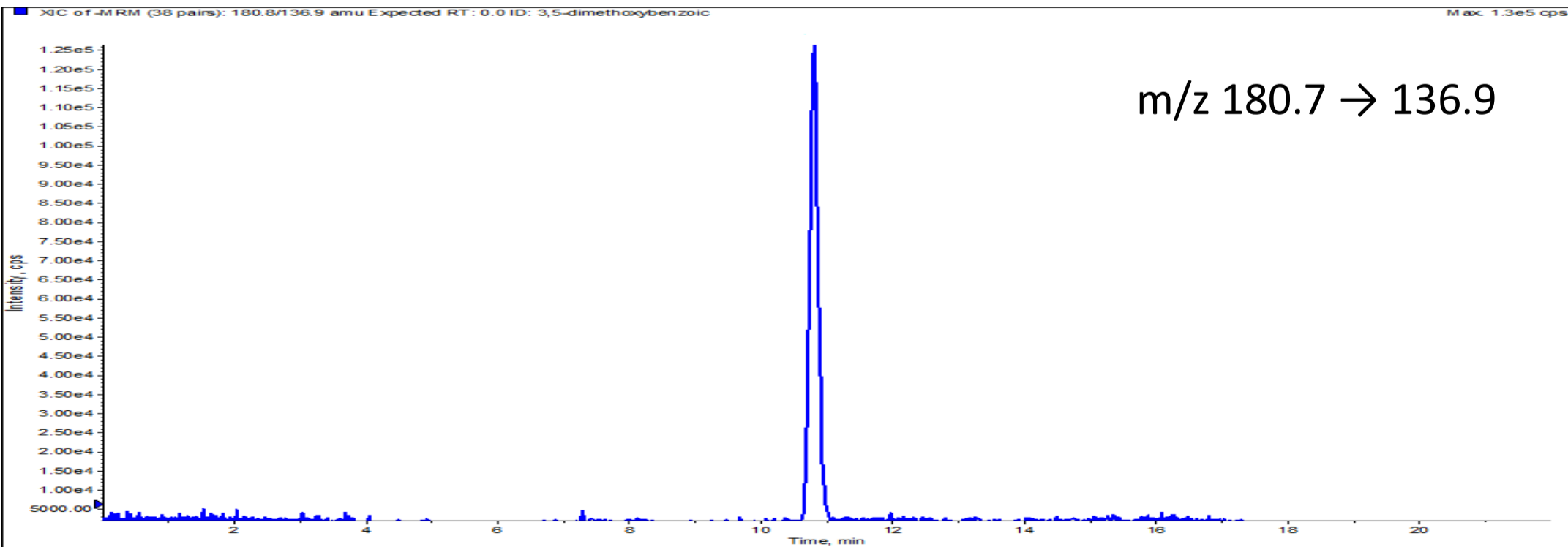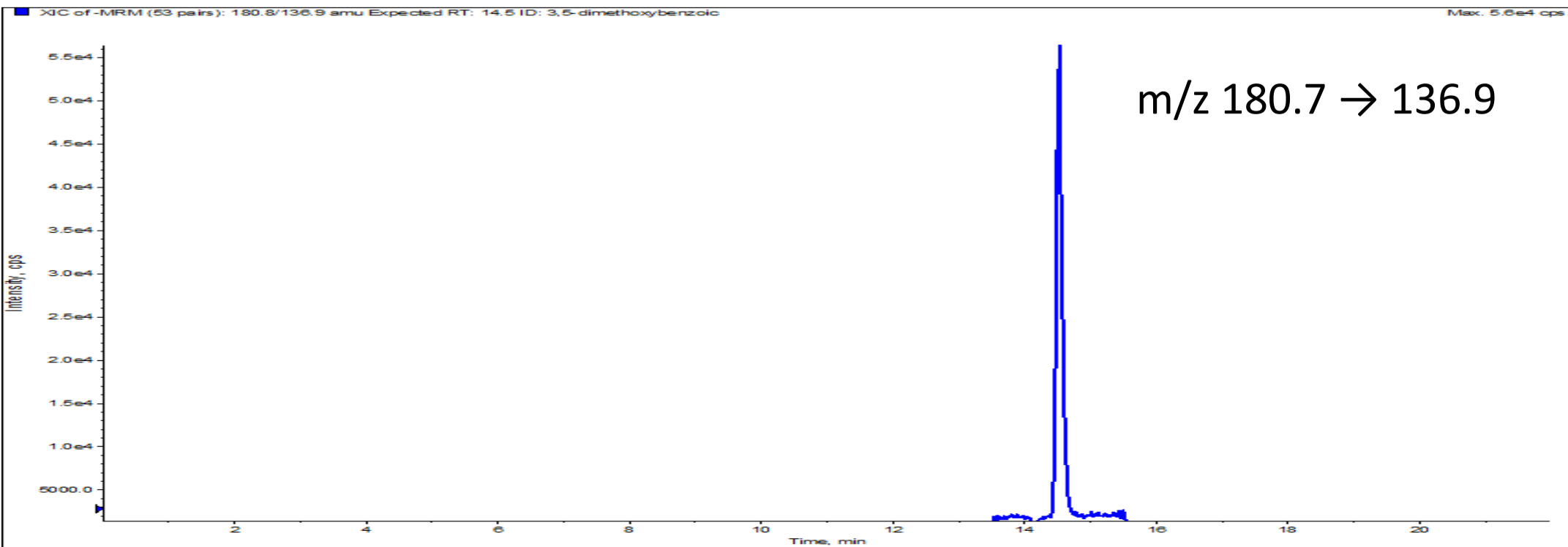

Supplement: Supplementary file 1 [file molecules-25-01804-s001.zip › molecules-765063-supplementary/Figure S1.pdf]

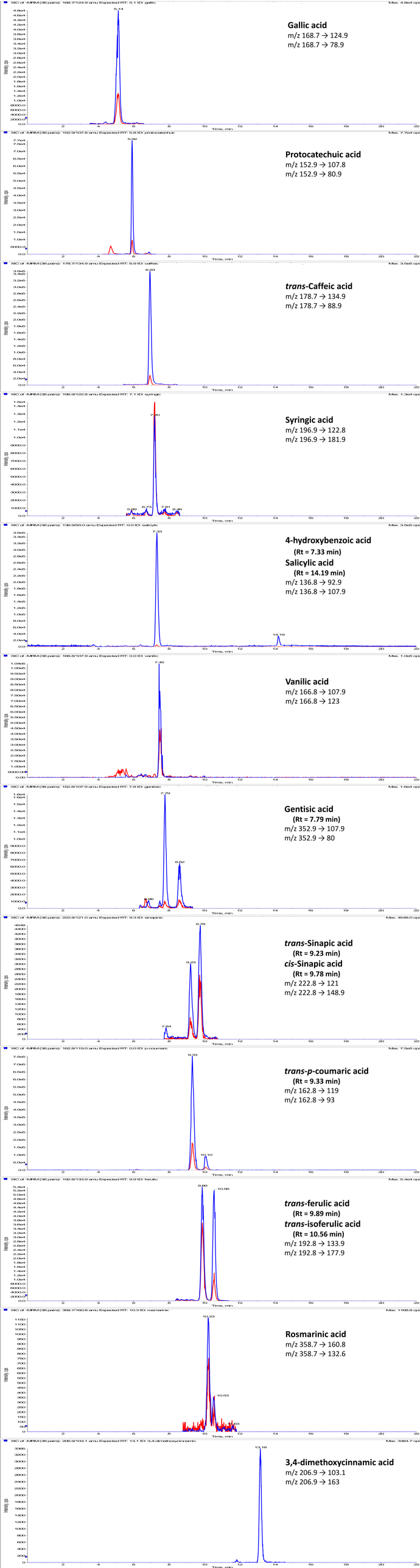

Supplement: Supplementary file 1 [file molecules-25-01804-s001.zip › molecules-765063-supplementary/Figure S2.pdf]
